# Nocturnal peak in cardiac baroreflex effectiveness aligns with nocturnal trough in systolic blood pressure in young healthy adults: Possible circadian effects

**DOI:** 10.14814/phy2.71092

**Published:** 2026-09-02

**Authors:** Leandro C. Brito, Megan L. Jones, Nicole Chaudhary, Steven A. Shea, Saurabh S. Thosar

**Affiliations:** ^1^ Oregon Institute of Occupational Health Sciences Oregon Health and Science University Portland Oregon USA; ^2^ School of Nursing Oregon Health and Science University Portland Oregon USA; ^3^ OHSU‐PSU School of Public Health Oregon Health and Science University Portland Oregon USA; ^4^ Knight Cardiovascular Institute Oregon Health and Science University Portland Oregon USA

**Keywords:** autonomic nervous system, blood pressure, circadian rhythm, sleep, vascular endothelial function

## Abstract

We tested whether there is a circadian variation in baroreflex function measured as sensitivity and effectiveness, and whether it aligned with circadian variations in systolic blood pressure (BP). Eleven healthy adults (24 ± 2 years; 4 males and 7 females) completed a 30‐h circadian protocol incorporating five recurring, identical, 6‐h cycles of 4‐h standardized wakefulness and 2‐h sleep opportunities. Spontaneous beat‐to‐beat BP (finger photoplethysmography) and R‐R intervals (electrocardiogram) were recorded at rest for 10 min during each wakefulness period, to assess cardiac baroreflex sensitivity (BRS) and effectiveness index (BEI). Vascular endothelial function (VEF) was assessed as flow‐mediated dilation of the brachial artery. Dim light melatonin onset was used as a circadian phase marker. Systolic BP exhibited a significant trough at ~1:40 am (*p* = 0.02). This trough closely aligned with the nocturnal peak of cardiac BEI that occurred at ~1 am (*p* = 0.02). BRS showed no significant variation. VEF exhibited a significant peak at ~10 am In healthy adults, the nocturnal peak in BEI is possibly aligned with the circadian trough in systolic BP. It remains to be determined whether shifting one would shift the other in a similar direction, and whether the alignment holds in people with disease.

## 1 INTRODUCTION

Blood pressure (BP) is usually lower at night than during the day, a pattern known as “nocturnal BP dipping” (Fagard et al., 2009; Hansen et al., 2006).

While the often observed changes in BP across the day and night are likely influenced by the daily behavioral cycles, such as sleep/wake, rest/activity, and fasting/feeding cycles (Bohmke et al., 2026; Jones et al., 2006; Thosar et al., 2025), it has been shown that there is also an underlying endogenous circadian rhythm in BP that occurs independent of daily behavioral cycles in healthy individuals (Hu et al., 2011; Scheer et al., 2010; Shea et al., 2011; Thosar et al., 2019). Across those studies, this endogenous BP rhythm was found to peak consistently in the evening, and to have troughs throughout the night or in the early morning. Interestingly, in these studies, only the autonomic nervous system showed potential to contribute to the circadian phase of BP regulation. For instance, the lowest plasma levels of norepinephrine (i.e., sympathetic neurotransmitter) occurred ~4 am, while the greatest plasma levels varied between 1 and 5 pm (Hu et al., 2011; Scheer et al., 2010). Adjustments in BP caused by the autonomic nervous system are mediated in the central nervous system mainly by the baroreflex system, either via cardio‐vagal adjustments in heart rate (Mancia et al., 1978) or via sympathetic nervous system influences on vascular resistance (Grassi et al., 1998). Thus, it is possible that the baroreflex system could be involved in the circadian rhythm of BP.

Previous studies have assessed cardio‐vagal baroreflex sensitivity across the 24 h behavioral cycle (i.e., with sleep at night and wakefulness plus physical activity during the day) in people with normal and high BP, and found diurnal variations with greatest cardio‐vagal baroreflex sensitivity during the night (~3 am) and lowest sensitivity in the morning (~8 am) (Hossmann et al., 1980; Parati et al., 1988; Tochikubo et al., 1997). The presence of a diurnal variation suggests that a potential circadian variation in baroreflex function is possible. However, this has not been tested using a validated circadian protocol that controls for the 24‐h behavioral sleep/wake, resting/activity, and fasting/feeding cycles. Thus, we studied whether there is a possible circadian variation in baroreflex function measured as sensitivity and effectiveness, and if existent, whether circadian variation in baroreflex function aligns with circadian variations in BP. We achieved this by conducting a “forced desynchrony” (FD) protocol in healthy young adults to assess circadian variation while controlling for daily behavioral/environmental influences (Brito et al., 2026).

## 2 METHODS

These data are a secondary analysis of a study investigating whether the circadian system modulates BP, hemodynamic determinants, and skeletal muscle blood flow changes following exercise, with one additional participant compared to what was previously published (Brito et al., 2026). The study was approved by the Institutional Review Board for the protection of Human Subjects at Oregon Health & Science University (OHSU) (IRB 24564). All participants signed a written informed consent prior to participation.

### 2.1 Participants

Volunteers were recruited using flyers posted around the OHSU campus and the Portland metropolitan area, word of mouth, research volunteer databases, and internet recruitment advertisements.

We recruited healthy individuals aged 20–39 years who were free of chronic physical or psychological conditions and who had not worked on night shifts in the prior 12 months; free of tobacco, and were not pregnant. Additional eligibility included having a resting BP <130 mmHg systolic and <80 mmHg diastolic (Whelton et al., 2018), body mass index >18.49 and <30.00 kg/m^2^, and physical activity status not more than recreationally active (<300 min/week of moderate‐intensity or <60 min/week of vigorous intensity) (Craig et al., 2003; Hallal & Victora, 2004). These criteria were used to keep our sample somewhat homogeneous and to avoid confounding effects of other factors on the cardiovascular circadian rhythms. Resting BP was determined during a screening visit after 10 min of seated rest by measuring BP three times on each arm using an oscillometric device (Dinamap, GE Medical Systems, Milwaukee, WI). The average value from all measures determined resting BP at screening. Habitual physical activity levels were determined based on the short version of the International Physical Activity Questionnaire (IPAQ) (Hallal & Victora, 2004).

### 2.2 Study design

To stabilize sleep and circadian rhythmicity, participants were instructed to follow a self‐selected 8‐h in‐bed schedule for at least 7 days immediately preceding the FD protocol. The adherence to these instructions was confirmed by participants filling out a sleep diary and calling a time‐stamped mailbox to report their wake and bedtime. Participants were instructed to refrain from consuming alcohol, non‐prescription drugs, caffeine, and exercise for 24 h prior to admission for both visits, which was verified by self‐report. All female participants were tested during the early follicular phase of their menstrual cycle. On admission, the absence of pregnancy in female participants (Pregnancy tests (HCG), Easy@Home, Burr Ridge, IL), and the absence of recreational and opiate drugs (Drugsmart 12 panel cup; Speares Medical, Inc., SC), and nicotine (NicAlert; Nymox Corporation, NJ), were confirmed by urinalysis.

#### 2.2.1 Forced desynchrony protocol

**FIGURE 1:**
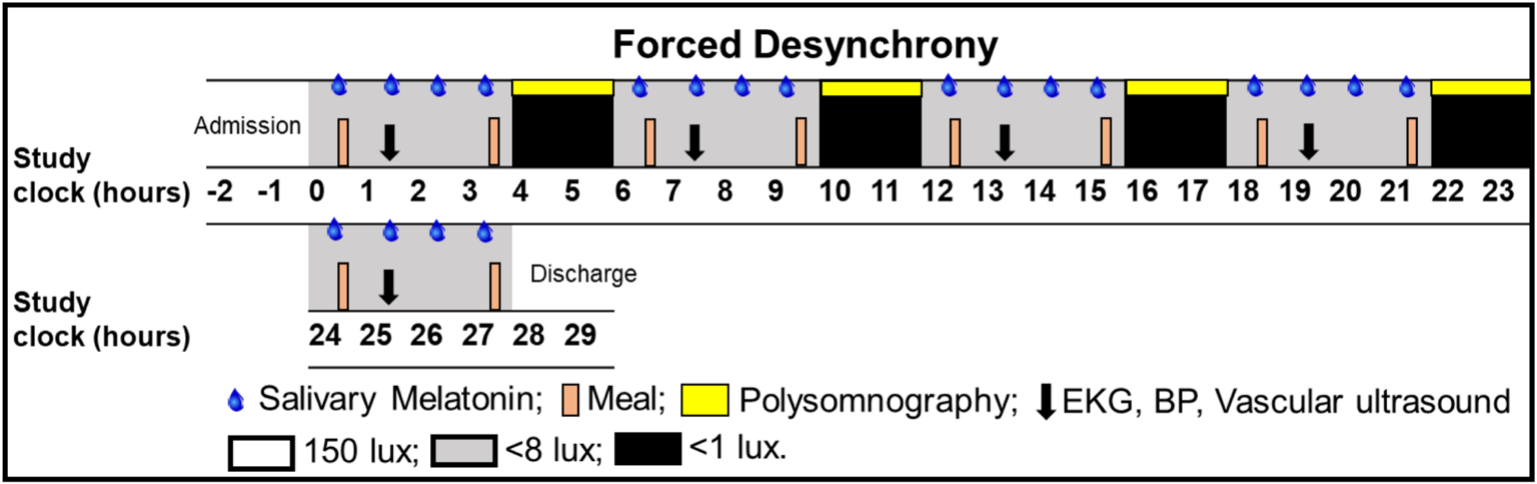
Forced desynchrony protocol. Participants completed a 30‐h protocol, blinded to the time. After 2 h of admission period, all participants underwent five consecutive 6‐h days (i.e., 4 h wake period and 2 h of sleep opportunity). White bars represent room lighting at 150 lux; black bars, sleep opportunities in total darkness (<1 lux); and gray bars, dim light wake periods below 8 lux. Blue drops represent salivary sampling, black upside‐down arrows represent measurements (EKG, beat‐to‐beat blood pressure, and vascular ultrasound), and brown bars represent isocaloric meals.

The protocol consisted of a 30‐h FD in‐lab stay, during which the first 2 h were used for participants' familiarization with all measuring processes, as previously published (Figure 1) (Brito et al., 2026). The FD protocol (Kleitman, 1939) uncovers underlying circadian rhythmicity in physiological functions by desynchronizing the sleep–wake cycle's effect from the effects of the endogenous circadian system. This is achieved by using a behavioral cycle length outside the limit of entrainment of the human circadian pacemaker (Czeisler et al., 1999; Dijk & Czeisler, 1994; Shea et al., 2011) and enables the circadian system to ‘free run’ at its inherent rate of ~24.1 h (Duffy et al., 2011; St Hilaire et al., 2012). To avoid any effects of light exposure on the circadian system (Boivin et al., 1996) during the FD protocol, wake periods were conducted in dim light (<8 lux) and sleep opportunities in darkness (<0.1 lux). Throughout the FD protocol, no time cues were provided to participants. Room temperature was controlled throughout the study at 22°C.

We maintained a 2:1 wake: sleep ratio in the FD protocol as previously published (Brito et al., 2026; Qian et al., 2020; Thosar et al., 2023). This was achieved via five identical recurring 6‐h behavioral cycles, comprising 4 h of wakefulness immediately followed by 2 h of sleep. Participants consumed an identical iso‐caloric meal every 3 h during the FD, with meals designed and prepared by a bionutritionist to match each participant's 24‐h total caloric need based on their body composition and self‐reported level of habitual physical activity. On average, caloric consumption per meal was 322 kcal with a range of 238–425 kcal. Each meal contained ~35% fat, ~50% carbohydrate, and ~15% protein. Polysomnography was used to continuously monitor sleep during each sleep opportunity (SOMNOtouch RESP, SOMNOmedics America Inc., FL, USA). At the beginning of each wake period, participants were gradually awakened using a standardized mild‐intensity auditory tone, and the lights were turned from darkness (<0.1 lux) to dim (<8 lux). Participants had all activities and posture tightly controlled, remaining supine on the bed except for exercise, scheduled restroom visits, and mealtimes. Measurements started 40 min after awakening during each wake period. Beat‐to‐beat BP and EKG were continuously recorded for 10 min, followed by assessment of vascular endothelial function (VEF) using ultrasound imaging of the brachial artery. The exact sequence of measurements was repeated every waking period. Once the FD protocol was completed, participants could choose between recovery sleep followed by discharge or pre‐arranged transportation home.

### 2.3 Measurements

#### 2.3.1 Circadian phase

Circadian phase was measured using the dim light melatonin onset (DLMO) (Benloucif et al., 2008) with melatonin assayed from salivary samples collected hourly during wake periods as previously described (Brito et al., 2026; Thosar et al., 2019). In brief, saliva was collected using a Salivette cotton swab (Sarstedt, Inc., NC) and immediately kept on ice to prevent degradation. Afterward, saliva samples were centrifuged at 1000 *g* for 2 min and aliquoted into 1000 μL portions for storage at −80°C until analysis. The DLMO was defined as the linearly interpolated time‐point at which melatonin crossed 3 pg/mL (Benloucif et al., 2008).

#### 2.3.2 Blood pressure and heart rate

We assessed cardiac R‐R interval using a standard three‐lead EKG. Beat‐to‐beat BP was measured using photoplethysmography with a cuff positioned on the participant's middle finger of the non‐dominant arm (NOVA Finometer, Finapres Medical Systems, Amsterdam, the Netherlands), which has been previously validated against intra‐arterial measurements (Parati et al., 1989). Cardiac R‐R interval and BP were recorded through a data acquisition system (Labchart version 8.0; AD Instruments). For statistical analyses, we used the average value from the 10‐min recording period.

#### 2.3.3 Cardiovascular autonomic modulation

The signals of R‐R intervals from EKG were recorded through an acquisition system (LabChart version 8.0; AD Instruments) using a sampling rate of 1000 Hz/channel. Data were exported to analysis software (CardioSeries v. 2.4, São Paulo, Brazil) to analyze stationary segments in parallel to a sinus respiratory rate measured using a thoracic piezoelectric belt (Respiratory belt sensor, ADInstruments). Stationarity was determined visually. Sympathetic vasomotor modulation was assessed by the absolute value of the low‐frequency band of systolic BP variability (LF_SBP_) (Pagani et al., 1997). Cardio‐vagal baroreflex sensitivity (BRS) was evaluated using the sequence technique as previously described (Parati et al., 1988). This method involves identifying three or more consecutive cardiac cycles in which systolic BP changes in the same direction (i.e., increases or decreases) by at least 1 mmHg while the R‐R interval changes in the opposite direction by at least 4 ms, and achieving a coefficient of correlation >0.80. The sensitivity was computed as the slope of the regression line relating changes in systolic BP to changes in R‐R interval. At the end, the slopes of the valid baroreflex sequences were averaged as follows: average of increases termed “up‐up sequences” (i.e., increase in R‐R intervals in response to an increase in systolic BP); average of decreases termed “down‐down sequences” (i.e., decrease in R‐R intervals in response to a decrease in systolic BP); and the average of all slopes (Parati et al., 1988). Cardio‐vagal baroreflex effectiveness index (BEI) was calculated as the ratio of the number of validated baroreflex sequences obtained using the sequence technique to the total number of systolic BP sequences, as previously described (di Rienzo et al., 2001). Cardio‐vagal BEI was calculated for up‐up, down‐down, and all sequences.

#### 2.3.4 Vascular endothelial function (VEF) and conductance of the brachial artery

To test potential macro and microvascular mechanisms explaining the circadian variation in BP, VEF was measured as flow‐mediated dilation following the instructions in the current expert consensus and the procedures in previous publications by our group (Thijssen et al., 2019; Thosar et al., 2018; Thosar et al., 2019). An image from the brachial artery was obtained 3–5 cm proximal to the antecubital fossa. Blood flow velocity (*V*_*b*_) and artery diameter (*D*_*v*_) were measured in both arteries by 1 min of duplex ultrasonographic recording using a linear‐array ultrasound transducer (L12‐4 probe, MindRay M9, CA, United States) with an insonation angle of 60°. Blood flow velocity and artery diameter were determined by automated edge‐detection software (Cardiovascular Suite, Quipu, Pisa, Italy). Blood flow was calculated as the following equation: Blood flow (mL/min) = $V_{b}\cdot 60\cdot {\left(D_{v}\cdot 2^{-1}\right)}^{2}\cdot \pi$. Vascular conductance (VC) was calculated by dividing the arterial blood flow by mean BP (mL/min*mmHg^−1^). The same trained evaluator analyzed all the ultrasound images while blinded to participant's number, date, and time of the image. Hyperemic shear was defined as the area under the curve of arterial shear rate from the time of deflation to peak diameter. Arterial shear rate was calculated from the following formula: $\mathrm{Vm}\cdot D^{-1}$, where *Vm* is mean blood velocity (cm·s^−1^) and *D* is mean arterial diameter (cm).

#### 2.3.5 Sleep

Sleep was monitored using polysomnography (SOMNOtouch RESP, SOMNOmedics America Inc., FL, USA) during the FD protocol, following the guidelines recommended by the American Academy of Sleep Medicine (Voinescu et al., 2014). After acquisition, the data were transferred to an automated analysis software package (Domino, SOMNOmedics America Inc., FL, USA) to quantify sleep, as previously reported by our group (Brito et al., 2026).

### 2.4 Statistical analysis

We employed a mixed‐model cosinor analysis to test for circadian variations of BP, cardiovascular autonomic variables, and vascular function assessed throughout the FD protocol. Our model consisted of circadian phase and time into the experiment as fixed factors. Participants were entered as a random factor and thus assigned a random intercept in the model. Specifically for cardio‐vagal BRS and BEI models, no statistically significant effect was observed when either the total number of systolic BP sequences or the number of validated baroreflex sequences was included as a covariate in the model. Thus, final cardio‐vagal BRS and BEI models plotted do not include these covariates. The DLMO was assigned as the circadian phase of 0°, and the circadian period was assumed to be 24.1 h (Duffy et al., 2011), as previously used by our group (Brito et al., 2026; Shea et al., 2011). Using this marker phase 0° and this assumed circadian period, it was possible to assign circadian phases between 0° and 359° to all other physiological data. The fundamental (~24 h) and second (~12 h) harmonics were included in the cosinor analysis of each variable to adjust for other frequencies in addition to a proper sinusoidal rhythm to determine a significant circadian phase against a null/non‐rhythm model. Circadian phases of peaks and troughs in these physiological variables were determined based on the cosine model plotted using the coefficients found in the mixed‐model cosinor analysis, with significance set at *p* < 0.05. For ease of interpretation, the time of day was estimated from the average DLMO time (clock time).

A one‐way ANOVA was used to assess the total number of systolic BP sequences and the number of validated baroreflex sequences across each wake period.

We performed additional analysis to explore the contribution of up‐up and down‐down sequences to the circadian effect of cardio‐vagal BEI. For this, we binned all cardio‐vagal BEI data (up‐up and down‐down sequences) into three 8‐h circadian phases based on each participant's DLMO time. For instance, the 8‐h window directly after DLMO was defined as circadian night, followed by circadian morning, and finally, afternoon. Then, we employed a one‐way repeated‐measures ANOVA with circadian phase (Morning, Afternoon, and Night) as the main factor. When the *p* value of the ANOVA test was <0.05, we performed a post hoc test with Bonferroni correction. Data are shown as mean ± SD with significance set at *p* < 0.05.

## 3 RESULTS

Eleven participants completed our 30‐h FD protocol, and their characteristics are shown in Table 1. On average, participants slept for a cumulative 7 h and 34 min throughout the 8 h of sleep opportunity across the entire FD protocol. No statistically significant differences were observed in sleep duration across 2‐h sleep opportunities (*p* = 0.50), with participants sleeping an average of 114 min (95% CI, 106–120 min). The time into protocol was not statistically significant for the cosinor model of any variable (all *p* > 0.05).

**TABLE 1:** Characteristics of the participants.

Variables	
Male/Female (*n*)	4/7
Race	
White (*n*)	7
Asian (*n*)	2
American Indian (*n*)	1
Unknown (*n*)	1
Ethnicity	
Hispanic or Latino	2
Non‐Hispanic or Non‐Latino	9
Age (years)	24 ± 2
Height (cm)	171 ± 11
Weight (kg)	71.8 ± 10.1
Body mass index (kg/m^2^)	24.5 ± 2.6
Systolic blood pressure (mmHg)	115 ± 8
Diastolic blood pressure (mmHg)	65 ± 6
Heart rate (bpm)	69 ± 10
Average bedtime at home (hh:mm)	22:48 ± 00:31
Average wake time at home (hh:mm)	06:54 ± 00:33
Dim light melatonin onset (hh:mm)	20:48 ± 00:45

*Note*: Values are shown as mean ± standard deviation or *n* (number of participants). Blood pressures were measured in triplicate in the office during screening visits. Hour: minutes (hh:mm). Average bedtime and wake time refer to the time reported by participants in a sleep diary.

**FIGURE 2:**
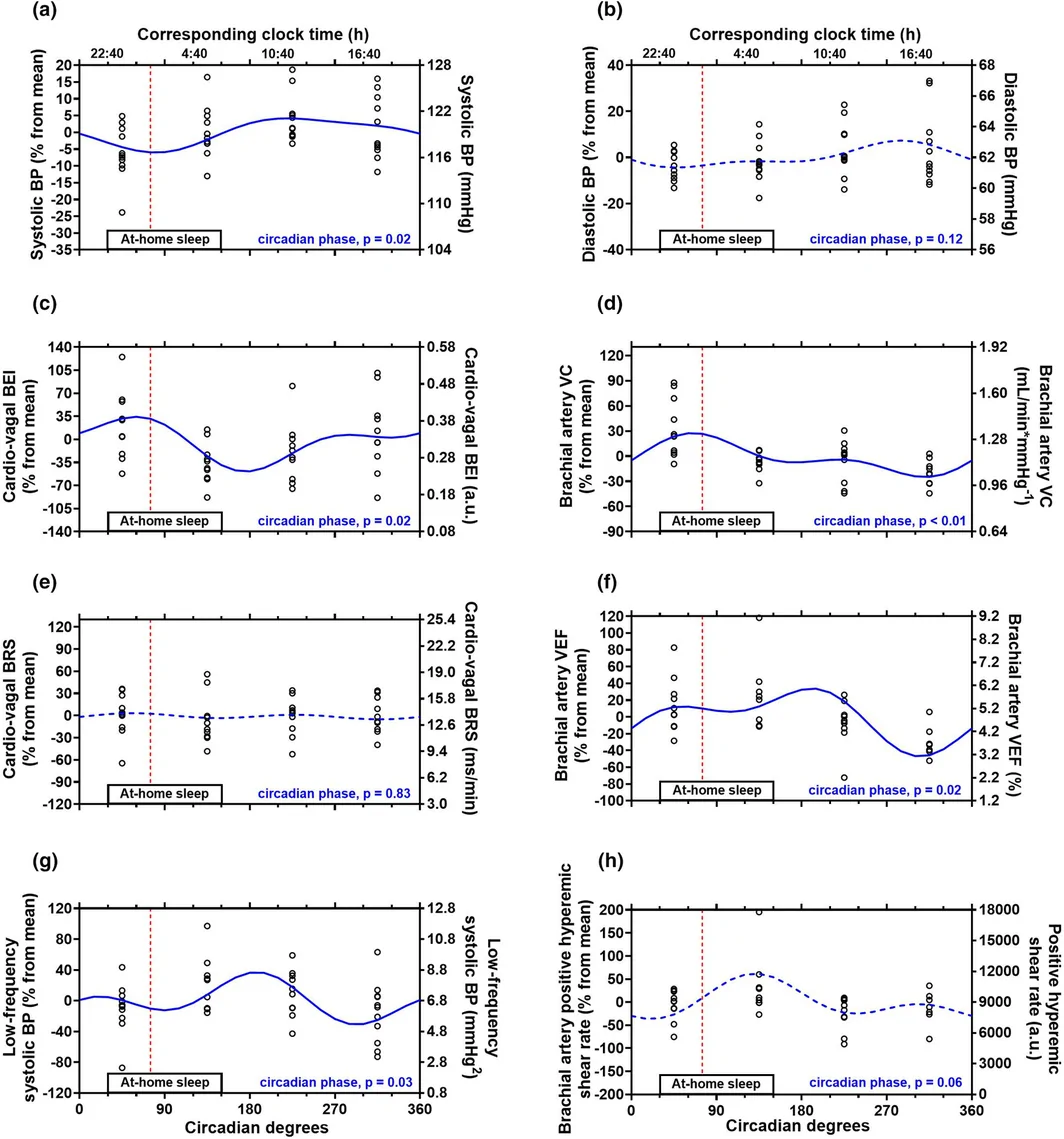
Circadian variation on resting absolute systolic blood pressure (BP) (a) and diastolic BP (b), cardio‐vagal baroreflex effectiveness index (BEI) (c), brachial artery vascular conductance (VC) (d), cardio‐vagal baroreflex sensitivity (BRS) (e), vascular endothelial function (VEF) (f), low‐frequency of absolute systolic BP variability (g), and brachial artery positive hyperemic shear rate (h). Models plotted with continuous lines are significant (*p* < 0.05), while dashed lines are not statistically significant (*p* > 0.05). The dashed red vertical line in all graphs indicates the trough of systolic BP based on the model. Individual data are shown plotted in 90° circadian bins and aligned with 0° representing the dim light melatonin onset, and habitual sleep represents the average clock time for the self‐selected sleep period at home (bottom X axis). Top X axis has clock time.

Our analysis revealed a significant circadian variation in systolic BP, with peak‐to‐trough change of 10 mmHg (*p* = 0.02, Figure 2a), and with the trough occurring at the circadian phase of 75°, corresponding to ~1:40 am, and a peak at 225°, corresponding to ~11:40 am. There was no significant circadian variationin diastolic BP (*p* = 0.12, Figure 2b).

Local hemodynamic and vascular functions across the circadian cycle also showed significant circadian variations. Brachial artery vascular conductance had the greatest conductance on average 27% above the mean at a circadian phase of 75°, corresponding to ~2 am, and the lowest conductance on average 25% lower than the mean at a circadian phase of 315°, corresponding to ~6 pm (*p* < 0.01, Figure 2d). Brachial artery VEF peaked much later, with the greatest function on average 34% above the mean at a circadian phase of 195°, corresponding to ~10 am, and the lowest function on average 47% lower than the mean at a circadian phase of 315°, corresponding to ~6 pm (*p* = 0.02, Figure 2f). Hyperemic shear used as a surrogate for microvascular function did not show a circadian variation.

**FIGURE 3:**
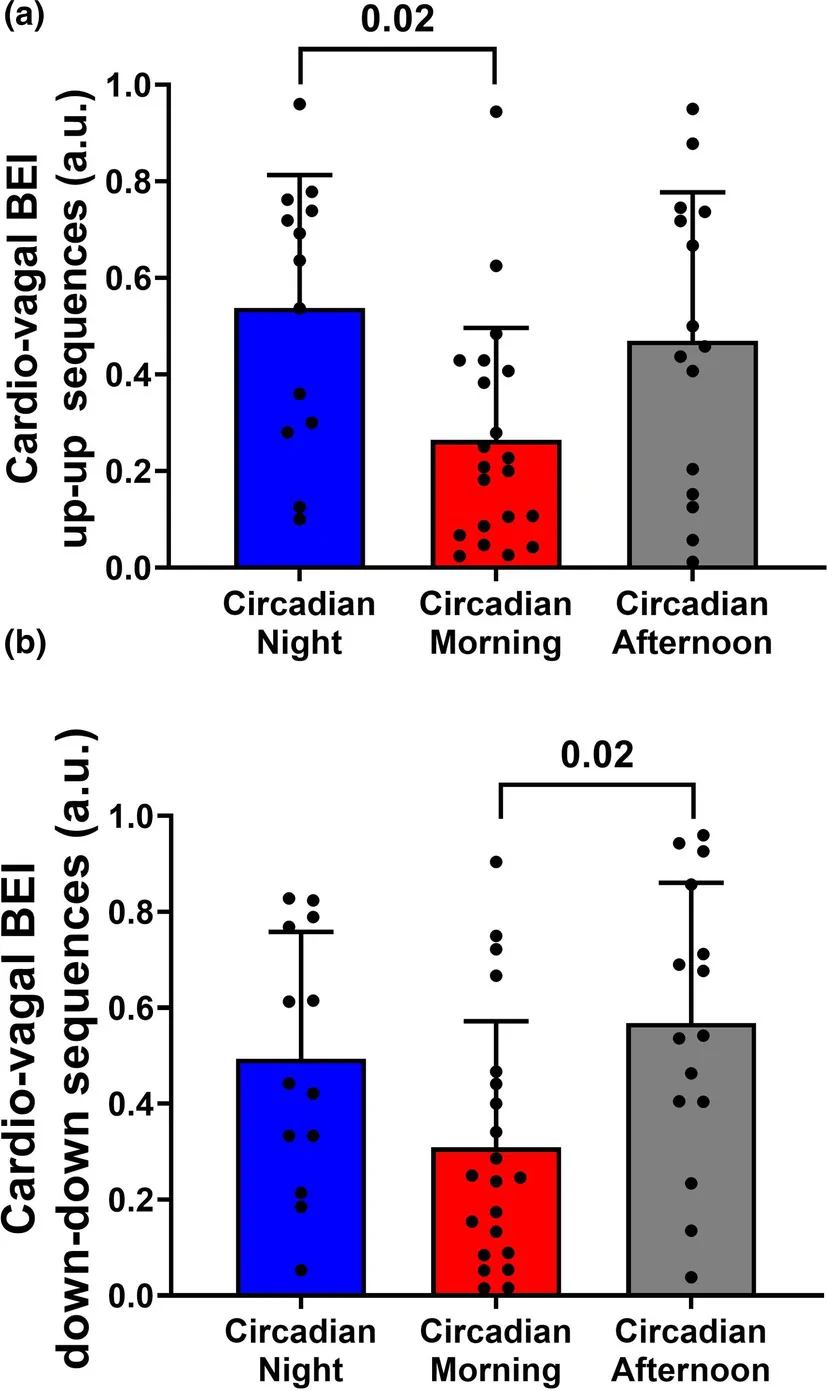
Circadian variation on up‐up and down‐down sequences of cardio‐vagal baroreflex effectiveness index (BEI) (a, b). Data were plotted for each participant into three 8‐h circadian phase bins representing circadian morning, afternoon, and night. To calculate the 8‐h bin, we defined circadian night based on each participant's dim‐light melatonin onset time. Bars represent mean values, and error bars represent standard deviation. Brackets indicate *p*‐value from multiple pairwise comparisons after a significant interaction in the one‐way ANOVA for repeated measures.

The spontaneous variations of cardiovascular autonomic mechanisms responsible for BP control did not reveal a circadian variation for cardio‐vagal BRS. On the other hand, cardio‐vagal BEI had a significant circadian variation, with the greatest effectiveness on average 34% above the mean at a circadian phase of 60°, corresponding to ~1 am, and the lowest effect on average 48% lower than the mean at a circadian phase of 180°, corresponding to ~9 am (*p* = 0.02, Figure 2c). Low‐frequency systolic BP showed a significant circadian variation, with the greatest sympathetic vasomotor modulation on average 36% above the mean at a circadian phase of 195°, corresponding to ~10 am, and the lowest effect on average 30% lower than the mean at 300°, corresponding to ~5 pm (*p* = 0.03, Figure 2g). Cardio‐vagal BEI up‐up sequences were significantly different only between circadian night and morning after pairwise comparisons by 110% (*p* = 0.02, Figure 3a), while cardio‐vagal BEI down‐down sequences were significantly different only between circadian morning and afternoon after pairwise comparisons by 84% (*p* = 0.02, Figure 3b).

See Table 2 for details of all mechanistic indices.

**TABLE 2:** Circadian variation of the absolute vascular function and cardiovascular autonomic indices.

Variable	*p* value circadian phase	Trough circadian ^0^/clock hour	Peak circadian ^0^/clock hour	Amplitude peak‐to‐trough
Cardiac baroreflex effectiveness index (a.u.)	**0.02**	180/~9 am	60/~1 am	0.25 (a.u.)
Cardiac baroreflex sensitivity (ms/mmHg)	n.s.	315/~5 pm	60/~1 am	2.8 ms/mmHg
Low‐frequency systolic BP (mmHg^2^)	**0.03**	300/~5 PM	195/~10 am	4.9 mmHg^2^
BA vascular conductance (mL/min*mmHg^−1^)	**>0.01**	315/~6 pm	75/~2 am	0.7 mL/min*mmHg^−1^
BA vascular endothelial function (%)	**0.02**	315/~6 PM	195/~10 am	4%
BA positive hyperemic shear rate (a.u.)	n.s.	15/~10 pm	135/~6 am	9985 (a.u.)

*Note:* Bold *p* values are significant.

Abbreviations: a.u., arbitrary unit; BA, brachial artery; BP, blood pressure; n.s., not significant.

**FIGURE 4:**
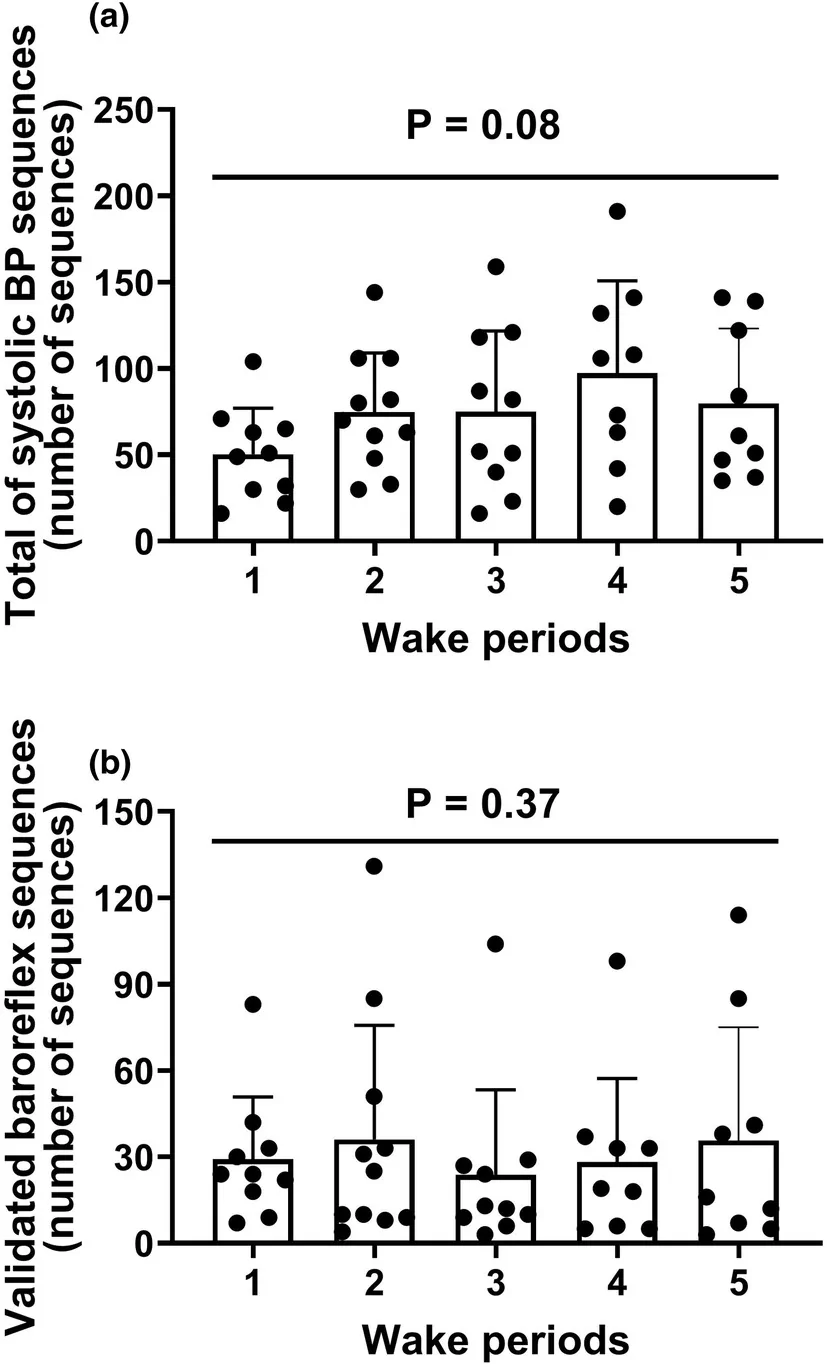
Average of the total number of systolic BP sequences (a) and average number of all validated baroreflex sequences (b) during each wake period across the forced desynchrony protocol. Data were plotted for each participant into the 5 wake periods. Bars represent mean values, and error bars represent standard deviation.

There was no statistically significant difference in the absolute total number of systolic BP sequences or validated baroreflex sequences. On average across each wake period, participants had 77 systolic BP sequences (95% CI, 55–89 sequences, *p* = 0.08, Figure 4a), and 30 validated baroreflex sequences (95% CI, 22–38 sequences, *p* = 0.37, Figure 4b).

## 4 DISCUSSION

We investigated the potential circadian variation of cardiovascular autonomic mechanisms and vascular function parameters, in particular microvascular function, that may contribute to BP control at rest. The main findings of the current study are (1) the nocturnal peak of cardio‐vagal baroreflex effectiveness possibly influenced by the circadian system; (2) the circadian trough of systolic BP occurs at a similar circadian phase as the greatest effectiveness of cardio‐vagal baroreflex and skeletal muscle vasodilation; and (3) the greatest brachial artery VEF function assessed by FMD occurred at the circadian phase corresponding to biological morning in healthy young adults.

We found a potential circadian variation in the spontaneous effectiveness of the cardio‐vagal baroreflex system. In addition, the greatest effectiveness of the cardio‐vagal baroreflex occurred at a circadian phase similar to the trough in systolic BP (i.e., ~1–2 am). Thus, it is reasonable to suggest that baroreflex effectiveness may influence the lowest BP values during the biological night, which could be at least partially influenced by the circadian system. This theory is reinforced by the fact that the cardio‐vagal baroreflex system was more effective at counteracting increases in BP but not decreases in BP during the circadian night (Figure 3a,b, respectively). In this respect, a preserved baroreflex function to correct increases in BP appears to weigh more than its ability to correct decreases in BP to control resting BP. This is clinically relevant. For example, it has been shown that, in women with a family history of hypertension, the baroreflex system has a reduced ability to correct increases in BP but preserved ability to correct decreases in BP (Matthews et al., 2019). In addition, in people with hypertension, the same deficiency of the baroreflex system in correcting BP increases, but not the correction of BP decreases, is independently associated with death and adverse cardiovascular events (Ormezzano et al., 2008).

Perhaps surprisingly, no significant circadian variation was found in the sensitivity of the baroreflex system. Previous diurnal studies have found greater BRS at nighttime (~3 am), but those results occurred in protocols where daily behaviors were not controlled across the day and night (Conway et al., 1983; Hossmann et al., 1980; Parati et al., 1988; Tochikubo et al., 1997). Previous diurnal studies either evaluated the maximal BRS using potent vasoactive drugs (i.e., modified‐Oxford technique) (Conway et al., 1983; Parati et al., 1988), or examined it in people with hypertension (Hossmann et al., 1980; Parati et al., 1988; Tochikubo et al., 1997). Although we did not directly investigate the underlying mechanisms of cardio‐vagal BRS, the discussion may encourage us and others to explore that path in future circadian protocols. All participants in the current study were healthy young adults who were also recreationally active. The high healthiness of our participants may have created a ceiling effect for cardio‐vagal BRS in the current study that masked a significant circadian variation. Previous studies conducted with healthy young individuals claimed a potential ceiling effect with no differences in BRS when employing maneuvers known for changing BRS sensitivity, such as managing respiratory rate (Gholamrezaei et al., 2021) or comparing healthy young adults who were sedentary, moderate‐trained, or highly‐trained in endurance (Monahan et al., 2000). Thus, differences in participants' characteristics or methodology may explain the absence of a circadian variation in BRS in the current study. It would be useful for future studies to extend our study by comparing individuals with different levels of resting BP, including people with hypertension. Another possible explanation for the discrepancy between BRS and BEI in the current study is that they act in counterphase, as shown by di Rienzo and colleagues when they proposed the BEI method (di Rienzo et al., 2001). Thus, it is possible that the circadian system had a stronger influence on the spontaneous effectiveness of the baroreflex system, attenuating its sensitivity in the current study. We note that the presence of a significant nocturnal peak observed in BEI or the absence of a significant variation in BRS occurred independently of the number of systolic BP sequences (Figure 4a) or the number of validated baroreflex sequences (Figure 4b) after they were included as covariates in the mixed‐model cosinor analysis. Considering that data were recorded for 10 min per measurement in the current study, the 30 validated baroreflex sequences observed (95% CI, 22–38) are consistent or even slightly higher than the previously reported expectation of about 80 sequences per hour in healthy adults (Parati et al., 1988). In fact, 80 validated baroreflex sequences per hour, divided by six 10‐min windows, would, on average, yield 13.3 sequences. Thus, the absence of a circadian variation in BRS or the presence of a significant circadian phase with a peak in BEI during the biological night should not be attributed to the number of validated baroreflex sequences observed.

The greatest VEF assessed by FMD occurred at the circadian phase corresponding ~10 am (*p* = 0.02; Figure 2f). Although the trough in systolic BP is not explained by the significant circadian phase in VEF found in our study, our data suggest a protective role of the circadian system over the cardiovascular system during the circadian morning. We found the greatest VEF ~5 h earlier in young adults than previously demonstrated in mid‐life adults (i.e., ~10 am vs. ~3 pm) when we used the same technique in a similar circadian FD protocol (Thosar et al., 2019). Notably, diurnal studies found lower VEF in the morning (6 am) compared to late morning (11 am) and evening (9 pm) (Otto et al., 2004). Our study may have relevance to the epidemiological findings that adverse cardiovascular events cluster in the morning between 6 am and noon (Muller et al., 1985). Thus, the greatest VEF at 10 am could suggest a protective role of the circadian system in healthy young adults. In fact, the aging process may affect the circadian system across various tissues (Barth et al., 2021), and could even affect cardiovascular risk biomarkers, such as BP reactivity to stressors (Thosar & Shea, 2025). Thus, it is reasonable to suggest that aging could affect the circadian rhythm in VEF, which should be systematically tested in the future.

The greatest vasodilation occurred in skeletal muscle immediately before the lowest systolic BP, as indicated by the greatest vascular conductance (Figure 2d). Although VEF did not explain the lowest systolic BP across the circadian cycle, microcirculatory function, as represented by hyperemic shear rate, showed a trend toward a possible circadian variation with a peak at ~6 am (*p* = 0.06) (Figure 2h). The secondary‐analysis nature of this study does not allow us to rule out the possibility that insufficient statistical power could explain the absence of a significant rhythm in microcirculatory function. Thus, it would be useful to reproduce our findings and, in future studies, explore whether microcirculatory mechanisms are associated with the circadian rhythm of vascular conductance and BP. Notably, local mechanisms involved in microcirculatory function, such as metabolic products (i.e., O_2_ and CO_2_ exchange and lactate) and the myogenic response of vascular smooth muscle (Hudlicka, 2011) have not yet been explored in the context of BP circadian variation. In addition, Sato and colleagues found that muscle metabolism and multiple muscle metabolites have a diurnal variation in sedentary rats (Sato et al., 2022). Consistent with our findings, a previous study did not find a potential contribution from the trough in sympathetic vasomotor modulation assessed by systolic BP variability aligned with the BP trough (Hu et al., 2011). However, plasma noradrenaline appears to occur at similar circadian phases (~4 am) to the systolic BP trough (Hu et al., 2011; Scheer et al., 2010). Thus, we cannot disregard a potential influence of the circadian rhythm of the sympathetic nervous system on the circadian lowest BP. Future studies using a direct assessment of sympathetic activity in humans (e.g., microneurography) could add specific perspectives on sympathetic baroreflex contribution to the circadian lowest BP.

The timing of the troughs of circadian variations in systolic BP in the current study differed somewhat from those observed in previous studies using similar circadian protocols in humans. Consistently, in four different circadian protocols – three FDs and one constant routine– systolic BP had a peak at the circadian phase corresponding to ~9 pm (Hu et al., 2011; Scheer et al., 2010; Shea et al., 2011; Thosar et al., 2019). However, the trough does not have the same consistency. Notably, the three forced FD protocols with behavioral cycle periods of wakefulness lengthening scheduled at less than 24 h had the lowest systolic BP during the circadian night at ~4 am (Hu et al., 2011; Scheer et al., 2010; Thosar et al., 2019), similar to the current study (~1 am). However, the FD protocol with a behavioral cycle of 28 h and the constant routine (40 h of continuous wakefulness) both found a trough for systolic BP around noon (Shea et al., 2011). The reasons for these differences are unknown and could be explored in future studies.

Further exploration of the current study's findings could be important for a more comprehensive investigation of the mechanisms underlying *non‐dipping* BP patterns. Previous studies have consistently demonstrated a diurnal pattern of the baroreflex system in people with normal and high BP, and all the findings pointed to a greater function in the middle of the night (Conway et al., 1983; Hossmann et al., 1980; Parati et al., 1988; Tochikubo et al., 1997). Our findings suggest that baroreflex effectiveness (i.e., the number of times the baroreflex system successfully corrects BP) (di Rienzo et al., 2001) is highest in the middle of the night and possibly influenced by the circadian system. Notably, no anti‐hypertensive medication directly acts on the baroreflex system. However, non‐pharmacological therapies, such as exercise, respiratory muscle training, and non‐invasive vagal nerve stimulation, are well known for improving baroreflex function and could be tested in chronotherapy‐based interventions. Interpretation of these findings requires caution, as they should be reproduced in people with *non‐dipping BP*.

The current study has several strengths but also some limitations. The analysis of the spontaneous baroreflex allows evaluation of small BP variations, consistent with those that occur during sleep or rest at night (Leroy et al., 1996). In contrast, more invasive tests, such as the tilt test or drug infusion for the modified‐Oxford technique, limit analysis to baroreflex sensitivity only, not to effectiveness (di Rienzo et al., 2001; Hart et al., 2010; Hunt & Farquhar, 2005; la Rovere et al., 2008). Among the limitations is the generalizability of the results. Previous studies suggest that sex can influence circadian rhythms, such as DLMO, which may occur earlier in females than in males (Cain et al., 2010). Sub‐analysis in the current study did not reveal statistically significant sex differences (all *p* > 0.05). Notably, the study sample has an unequal sex distribution with small groups (males = 4 and females = 7), which limits the statistical power to detect sex differences. Future studies could build upon our work to test sex differences. In addition, aging and health characteristics, such as body mass index and resting BP levels, can affect baroreflex (Monahan, 2007) and vascular function (Holder et al., 2021). Thus, our results should not be generalized to individuals of different ages and clinical conditions (e.g., hypertension). However, our rigorous selection of healthy participants from a homogeneous sample enables us to derive mechanistic insights from our findings, opening new avenues for investigating nocturnal BP regulation. Notably, our FD protocol limits our resolution to 6 h, which overlaps with the 4th harmonic of a 24‐h period. This potentially affects peak and trough precision and likely underestimates the amplitude of endogenous circadian variation. In addition, our abbreviated FD protocol design with a total of 30 h could be masked by the behavioral cycles (i.e., the detected 24 h rhythms may reflect influence from the circadian clock or a combination of the circadian and behavioral cycles). On the other hand, we did not find a statistically significant effect of the time into protocol in the variables analyzed, which could be explained by the short duration of our FD protocol lasting 30 h. The trough of systolic BP is less consistent than the peak, and longer circadian protocols should be performed to confirm whether baroreflex effectiveness contributes to the nocturnal trough in systolic BP. Finally, circadian protocols have translational boundaries to a real‐world interpretation when concurrent and cumulative influences on the cardiovascular system occur due to ongoing environmental and behavioral factors. However, only highly controlled circadian protocols, as we have used, can uncouple the influence of behaviors and the environment from the control exerted by the endogenous circadian system.

In conclusion, in healthy young adults, our results suggest a possible circadian effect on spontaneous baroreflex effectiveness with a peak during the biological night. The cardiac baroreflex system appears to act most frequently during a circadian phase similar to the lowest nocturnal decline in systolic BP. This finding launches the baroreflex system as a promising candidate for studying the mechanisms underlying the non‐dipping pattern of BP. Future studies should investigate the circadian rhythm of the baroreflex system in the nocturnal BP decline in longer circadian protocols, including more frequent sampling to improve resolution.

## AUTHOR CONTRIBUTIONS

**Leandro C. Brito:** Conceptualization; data curation; formal analysis; funding acquisition; investigation; methodology; project administration. **Megan L. Jones:** Methodology; project administration. **Nicole Chaudhary:** Methodology; project administration. **Steven A. Shea:** Data curation; funding acquisition; resources; supervision. **Saurabh S. Thosar:** Conceptualization; data curation; funding acquisition; resources; supervision.

## FUNDING INFORMATION

This work was supported by the Medical Research Foundation of Oregon and American Heart Association Career Development Award 24CDA1267757 (to L.C.B.); National Institutes of Health R01‐HL163232 (to S.S.T.); NIH Grant R35‐HL155681 (to S.A.S.); the Oregon Institute of Occupational Health Sciences at Oregon Health and Science University via funds from the Division of Consumer and Business Services of the State of Oregon (ORS‐656.630); and UL1‐TR002369 (to OHSU).

## ETHICS STATEMENT

All participants signed an informed written consent before enrollment. The study was approved by the Institutional Review Board for the protection of Human Subjects at Oregon Health & Science University (OHSU) (IRB 24564).

## Data Availability

The data supporting this study's findings will be available from the corresponding authors upon reasonable request.
